# Supplementary material for: Identification of the area sampled by traps: A modelling study with tsetse
Source: PLoS Negl Trop Dis. 2023 Jan 27;17(1):e0010902. doi: 10.1371/journal.pntd.0010902 (PMC9910695; doi:10.1371/journal.pntd.0010902)
Supplement: S1 Text — Table A in S1 Text. Percentage of total catches that originated from within various lengths of sampled habitat at different durations of trapping, involving one trap operated alone, or a combination of three traps arranged at 1km intervals. The sampled lengths are centred on the position of the one trap or on the central trap of the three traps. (DOCX) [file pntd.0010902.s001.docx]

**Supplementary Materials: S1 Text**

**Application of the model to sampling in a linear habitat**

The modelling for the linear habitat was basically the same as for the two-dimensional habitat, except for the following two changes. First, the habitat was represented by a line of 71 cells, instead of a block of 71 x 71 cells. In consequence, it was most appropriate to study the *distance*, rather than the *area*, within which various percentages of the catch originated. Second, while it was assumed that the flies attempted to vacate each cell equally in all four orthogonal directions each day, those that tried to go perpendicular to the line of the habitat would encounter non-habitat when they reached the edge of the cell. Such flies were regarded as turning back to the centre of the cell, so that their resulting movement during the day was zero. Thus, the daily evacuation rate of the cells was 39.5%, with half of the evacuees going to the habitat cell in one direction along the line, and the other half going in the opposite direction. Whether any given fly moved on any one day, and the particular direction in which it moved on that day, were envisaged as unrelated to its movement on the next day. Hence, allowing that the cell width was 0.5km, the mean distance between the start and end points of daily movement was 197.5m, *i.e.,* half the mean daily movement in the two-dimensional habitat.

To exemplify the outputs of the linear model, some of the studies were made with a single trap located in the central cell of the line of habitat cells. In other studies there were three traps positioned 1km apart, involving a trap in the central cell, and a trap 1km away in each of the two directions along the line. This meant that the outer traps were two cell widths away from the central cell. With each arrangement of traps, the trapping period ranged from 5-30 days, the catching efficiency, *E*, was 0.1, the natural daily death rate, *Mnat*, was 0.03, and there was no control mortality.

Table A in S1 Text shows the percentage of the total catch of the one trap, or of the three traps combined, that originated from various lengths of habitat centred on the central cell. Not surprisingly, the percentages coming from any given length of habitat decreased with an increase in the duration of trapping. For any given duration of trapping, the percentages increased with the length of habitat considered.

**Table A.** Percentage of total catches that originated from within various lengths of sampled habitat at different durations of trapping, involving one trap operated alone, or a combination of three traps arranged at 1km intervals. The sampled lengths are centred on the position of the one trap or on the central trap of the three traps.

| Sampled length | Duration of trapping | | | | | |
| --- | --- | --- | --- | --- | --- | --- |
|  | 5 days | 10 days | 15 days | 20 days | 25 days | 30 days |
| One trap alone | | | | | | |
| 1km | 85.56 | 74.54 | 68.01 | 63.71 | 60.67 | 58.41 |
| 2km | 97.57 | 91.77 | 86.93 | 83.23 | 80.38 | 78.15 |
| 3km | 99.75 | 97.78 | 95.20 | 92.79 | 90.72 | 88.98 |
| 4km | 99.99 | 99.51 | 98.43 | 97.14 | 95.87 | 94.69 |
| 5km | 100.00 | 99.92 | 99.55 | 98.97 | 98.28 | 97.57 |
| Three traps at 1km intervals | | | | | | |
| 1km | 68.01 | 46.24 | 44.29 | 42.70 | 41.44 | 40.44 |
| 2km | 86.93 | 74.27 | 70.78 | 68.19 | 66.19 | 64.64 |
| 3km | 95.20 | 90.55 | 87.21 | 84.62 | 82.59 | 80.96 |
| 4km | 98.43 | 97.01 | 94.89 | 93.03 | 91.45 | 90.12 |
| 5km | 99.55 | 99.21 | 98.16 | 97.06 | 96.02 | 95.09 |
