# Supplementary material for: Identification of the area sampled by traps: A modelling study with tsetse
Source: PLoS Negl Trop Dis. 2023 Jan 27;17(1):e0010902. doi: 10.1371/journal.pntd.0010902 (PMC9910695; doi:10.1371/journal.pntd.0010902)
Supplement: Attachment — Submitted filename: Covering letter 20221204 - version for reviewers.docx [file pntd.0010902.s003.docx]

4 December 2022

Dear Reviewers,

We are very grateful for your constructive reviews of our MS. We note that neither of you required changes to the substance of the MS, and called only for amendments to a few matters of presentation. We hope we have attended to those matters satisfactorily.

In responding to your comments, we did, nevertheless, go through the MS again to identify any possible points where less expert readers might benefit from minor rewording. We have made a few changes of that sort. None of such changes alter the sense of the text.

We submit two copies of the amended MS and two copies of its Supporting Information 2. One copy of each pair is a clean copy with the amendments, and the other copy of the pair employs yellow shading to show the changed sections. It might be difficult to spot the shading on the Supporting Information 1 document since it requires only two insertions of “a” – one in the second line of the first paragraph, and another in the second line of the caption of Table S1.

We have also provided an amendment of Supporting Information 1, which is the large Excel file that holds the programme and model on which the MS is based. You did not specifically ask for any amendments to this file but on checking through it ourselves we have found a few typos that required correction. The new file that we now provide contains the corrections, but since they are very minor, are hidden away in small corners of the file, and make no difference at all to the workings, outputs, and conclusions of the model, we have assumed that you will not require an additional file that gives account of the corrections.

On behalf of all of us, many thanks for your time and careful and constructive consideration

Yours faithfully,

Glyn, John and Steve

Prof. Steve Torr on behalf of all the co-authors
